# Responses to the AI Revolution in Hospitality and Tourism Higher Education: The Perception of Students Towards Accepting and Using Microsoft Copilot

**DOI:** 10.3390/ejihpe15030035

**Published:** 2025-03-14

**Authors:** Ahmed Mohamed Hasanein

**Affiliations:** Management Department, College of Business Administration, King Faisal University, Al-Hassa 31982, Saudi Arabia; aabdelrazek@kfu.edu.sa

**Keywords:** Microsoft Copilot, artificial intelligence, technology acceptance, technology usage, hospitality and tourism education

## Abstract

This research aims to examine hospitality and tourism students’ acceptance and usage of Microsoft Copilot for educational purposes in Egyptian public universities. It also investigates the mediating role of behavioral intention (BI) in the connection between hospitality and tourism students’ acceptance and actual use of Microsoft Copilot. This study adopted the unified theory of acceptance and use of technology (UTAUT) framework to achieve the research aim. A quantitative approach was used via online surveys distributed and gathered from 760 hospitality and tourism students from nine public universities in Egypt and analyzed using PLS-SEM to test the hypothesized relationships. The major findings showed that PE, EE, SI, and FC affected BI to use Microsoft Copilot and highlighted a substantial direct influence of SI, FC, and BI alone on the actual use of Microsoft Copilot. Therefore, BI partially mediates the relationship between SI and FC and real-world classroom utilization of Microsoft Copilot. This clarifies that BI has a slight role in the relationship between SI and FC and the actual use of Microsoft Copilot, while the relationship between PE, EE, and the actual use of Microsoft Copilot occurs entirely through BI. However, there was full mediation between the use of Microsoft Copilot in the classroom and BI between PE and EE. The results have several implications for Egyptian higher education institutions and academics of hospitality and tourism and are also relevant to other institutions in a comparable setting.

## 1. Introduction

Due to technological advancements in the use of AI tools in many different sectors, especially higher education (Popenici et al., 2023; Rudolph et al., 2023; A. M. Hasanein & Sobaih et al., 2023; bin Mohamed Ismail et al., 2024), hospitality and tourism higher education with a unique practical learning environment has received a considerable scholarly attention on students’ usage of AI platforms, e.g., Microsoft Copilot, for academic purposes (Popenici, 2023; Dogru et al., 2024). As a transformative ecosystem AI-driven tool, Microsoft Copilot plays a pivotal role in assisting students in their educational activities, such as generating text-like humans with the deep integration of Microsoft tools (Marquis et al., 2024). Within the groundbreaking collaboration between the Ministry of Higher Education in Egypt and Microsoft Corporation from the academic year 2020–2021, students have full access to Microsoft learning tools for their educational activities (Helmy, 2024). This partnership leverages AI-powered ftools to bridge the gap between theoretical learning and industry-specific skills, enabling students to access personalized assistance in course management, data analysis, and project development (Hamed, 2024).

Moreover, Microsoft Copilot’s real-time capabilities can streamline curriculum delivery, enhance language proficiency, and support digital transformation in institutions (Shamsudin & Hoon, 2024). However, the success of this initiative hinges on cohesive strategies, including faculty training, infrastructure modernization, and the alignment of AI functionalities (Hutson & Rains, 2024).

Numerous studies on the use of Microsoft Copilot in higher education (Chen, 2024; Nik Harun et al., 2024; Shumeiko & Osadcha, 2024; Adetayo et al., 2024) have revealed some beneficial features, including an immediate and efficient chatbot response to related or unrelated educational tasks, improving students’ language performance, and boosting their editing and proofreading skills. Another study by Chukwuere and Handoko (2024) claimed that Microsoft Copilot serves as an assistive tool for students in terms of their daily homework activities, assignments, and projects.

Despite this growth in the use of Microsoft Copilot in educational activities, there are increasing concerns raised by scholars, experts, and leaders in higher education regarding the misusage of Microsoft Copilot in educational activities, with a specific focus on learning and teaching skills (Nik Harun et al., 2024). These concerns encompass the potential biased information generated by Microsoft Copilot, inaccuracies, fake references, and reliability and intellectual property issues, which may raise ethical concerns regarding the misuse of this AI platform (Nikolic et al., 2024). Furthermore, overreliance on utilizing AI in educational contexts (e.g., Microsoft Copilot) and activities may negatively affect students’ critical thinking skills (Adetayo et al., 2024).

Numerous efforts have been made by researchers (Strzelecki, 2023; Shahsavar & Choudhury, 2023; Chen, 2024; A. Hasanein et al., 2024) to understand the use of AI in higher education (e.g., ChatGPT, Gemini), but there have been limited attempts to measure the perceptions of higher education students regarding the acceptance and usage of Microsoft Copilot in their educational settings (Nik Harun et al., 2024). A recent study by Chen (2024) explored Microsoft Copilot Studio with the integration of other AI tools such as ChatGPT to enhance student AI support, thereby fostering students’ AI motivation and engagement. To measure students’ perceptions regarding the acceptance and usage of AI platforms, research conducted by Strzelecki (2023) acknowledged a gap in research with reference to students’ acceptance and use of AI in higher education. Strzelecki (2023) adopted the UTAUT to measure the factors that influence students’ acceptance and use of AI, that is, ChatGPT in Polish higher education. The focus of this study was in line with this approach.

Drawing upon the UTAUT framework, this research explores students’ perceptions toward accepting and using Microsoft Copilot as a transformative AI tool for educational purposes in Egyptian hospitality and tourism higher education. Understanding the factors that influence students’ acceptance and use of Microsoft Copilot, which enables educational institutions and scholars to gain deeper insights into these drivers, provides educational leaders with the best practices for students’ utilization of AI, that is, Microsoft Copilot, in their educational activities. Ultimately, this study aimed to aid educational leaders in constructing effective guidelines that exploit the prospective utilization of AI to boost hospitality and tourism students’ learning experiences and facilitate educational achievements through their educational activities.

## 2. Literature Review and Hypotheses Development

### 2.1. Students’ Acceptance and Use of Microsoft Copilot and Behavioral Intention

To date studies (e.g., Anditama & Hidayanto, 2024; Kim et al., 2024; Strzelecki et al., 2024; Xia & Chen, 2024) have proved that the UTAUT model serves as a robust framework for exploring students’ acceptance and utilization of innovative AI platforms across varied contexts. Due to the adoption of Microsoft Copilot in educational contexts (Nik Harun et al., 2024; Shamsudin & Hoon, 2024), this framework provides a systematic approach for understanding students’ behavioral intentions (BI) regarding their use of these advanced means of AI (Strzelecki et al., 2024). As concluded by Venkatesh et al. (2003), there are several key factors that influence users’ perceptions regarding adopting and engaging with technology: effort expectancy (EE), performance expectancy (PE), social influence (SI), and facilitating conditions (FC). PE pertains to how students identify the ability of AI tools to boost their learning experiences (Shahsavar & Choudhury, 2023). As a result, students believe that innovative means of AI, such as Microsoft Copilot, may positively affect their learning experience and foster their BI (Pan et al., 2024; Menon et al., 2025). Another study by Anditama and Hidayanto (2024) claimed that students who believe that AI platforms, such as Microsoft Copilot, may assist them in their complicated modules, enhance their creativity, or cultivate their academic performance are more likely to express a positive BI toward using it in their educational activities. EE reflects users’ interactions with AI technology, considering their perception of the level of ease or difficulty of using AI (Yin et al., 2023; Kim et al., 2024). In addition, EE plays a crucial role in shaping students’ willingness to engage with AI tools such as Microsoft Copilot (Menon et al., 2025). Another study by Xia and Chen (2024) claimed that when students perceive Microsoft Copilot as accessible and intuitive, they tend to adopt it seamlessly in their daily academic activities. Recent studies (e.g., Adetayo et al., 2024; Nikolic et al., 2024; Nik Harun et al., 2024) have highlighted how factors such as ease of interaction with AI, platform design, and overall user experience influence students’ perceptions. When students expect little effort to use Microsoft Copilot, they are more likely to integrate it into their educational routines (Nik Harun et al., 2024).

SI refers to the influence of classmates and professors in shaping students’ attitudes toward Microsoft Copilot (Yin et al., 2023). Another study by Sobaih et al. (2023) revealed that SI is related to students’ perceived behaviors in terms of their integration with AI for learning purposes. Scholars have received great attention (e.g., Ma & Huo, 2023; Nik Harun et al., 2024; Pan et al., 2024; Menon et al., 2025) and have revealed that sharing a good experience of using AI in their learning activities will positively affect other students’ BI. FC measures the obtainability of the required AI tools for an effective use experience (Nikolic et al., 2024). Additionally, FC is related to educational institutions’ support through technological accessibility and training aids to AI tools, such as Microsoft Copilot (Adetayo et al., 2024). Menon et al. (2025) argued that when students perceive the needed guidance, help, and support from their educational institutions, it will positively affect their BI to use AI platforms, that is, Microsoft Copilot. Another study by Chukwuere and Handoko (2024) claimed that FC not only needed technological support but also related to generating technological infrastructure that facilitated students’ efficient navigation of Microsoft Copilot, thus boosting their BI toward using this innovative AI tool. Therefore, we propose the following hypotheses:

**H1.**  *PE positively influences students’ BI.*

**H2.**  *EE positively influences students’ BI.*

**H3.**  *SI positively influences students’ BI.*

**H4.**  *FC positively influences students’ BI.*

### 2.2. Students’ Acceptance to Use of Microsoft Copilot

Numerous recent studies (e.g., Schei et al., 2024; Tarek & Abd El Hamid, 2025) have emphasized the crucial role of EE and PE in mediating the relationship between students’ acceptance and use of AI tools. These pivotal elements are affected by various aspects of AI accessibility and ease of alignment with student needs (Nik Harun et al., 2024; Sobaih et al., 2024). Furthermore, some aspects are considered vital contributors to PE, such as achieving tasks, efficiency, and engagement (Ma & Huo, 2023). In the educational setting, Menon et al. (2025) claimed that PE is the foremost aspect that influences the perception of higher education students in terms of using AI platforms such as Microsoft Copilot in learning activities. Furthermore, Shamsudin and Hoon (2024) revealed that EE is related to students’ perceived ease of use, ease of access, and ease of implementation of AI tools, which may boost their experience in terms of value added and usage. Concerning SI, numerous studies (e.g., Adetayo et al., 2024; Hutson & Rains, 2024; Shamsudin & Hoon, 2024) have argued that positive feedback toward utilizing AI platforms via peers will positively affect students’ actual usage of AI tools, such as Microsoft Copilot. In terms of FC, a recent study by Kim et al. (2024) claimed that FC includes various aspects, such as the availability of technology infrastructure, immediate technical assistance, and guidance by educational institutions, which may affect students’ use of Microsoft Copilot. Based on this discussion, we hypothesize the following:

**H5.**  *PE positively influences usage of Microsoft Copilot.*

**H6.**  *EE positively influences usage of Microsoft Copilot.*

**H7.**  *SI positively influences usage of Microsoft Copilot.*

**H8.**  *FC positively influences usage of Microsoft Copilot.*

### 2.3. Students’ Behavioral Intention and Use of Microsoft Copilot

One of the key aspects of adopting and utilizing technology is the relationship between BI and AI tools, such as Microsoft Copilot, and actual usage. Based on a study by Venkatesh (2022), BI is the willingness and preparedness of people to interact with a specific technology. Many people believe that this is a direct prerequisite to the use of this technology (Shahsavar & Choudhury, 2023; Strzelecki, 2023). Several studies (i.e., Anditama & Hidayanto, 2024; Pan et al., 2024; Menon et al., 2025) have demonstrated that students are more likely to utilize Microsoft Copilot when they have a favorable BI toward it, indicating that they are prepared to use it in their daily routines or tasks. Additionally, the more students want to use Microsoft Copilot, the more likely they are to actively engage with the platform, ask for help, and incorporate it into their academic pursuits (Asag et al., 2024). Therefore, considering these factors, we propose the following hypothesis:

**H9.**  *BI has a positive direct influence on usage of Microsoft Copilot.*

### 2.4. The Role of BI in the Connection Between Students’ Acceptance and Use of Microsoft Copilot

**Figure 1:**
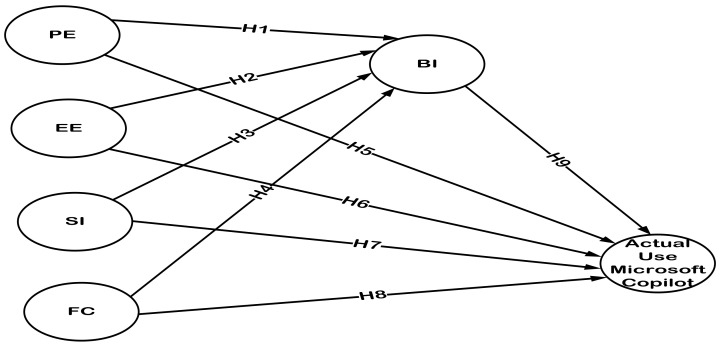
The Research Conceptual Model.

Based on the work of Adetayo et al. (2024), Chen (2024), Nikolic et al. (2024), and Menon et al. (2025), BI is essential to bridge the gap between students’ approval of Microsoft Copilot and its actual use in higher education settings. Nonetheless, there is a research gap regarding BI’s mediating function in this association. In particular, the current research is considered the first attempt to measure the mediating function of BI in the connection between students’ acceptance of Microsoft Copilot and its actual use in Egyptian hospitality and tourism institutions. By adopting the UTAUT framework, this study seeks to close this gap by applying the following theories (Figure 1 presents the research conceptual model).

**H10.**  *BI intermediates the connection between PE and the usage of Microsoft Copilot.*

**H11.**  *BI intermediates the connection between EE and the usage of Microsoft Copilot.*

**H12.**  *BI intermediates the connection between SI and the usage of Microsoft Copilot.*

**H13.**  *BI intermediates the connection between FC and the usage of Microsoft Copilot.*

## 3. Methodology

### 3.1. Research Design and Population

This study employed quantitative research design, using a cross-sectional survey approach. The study population comprised hospitality and tourism students enrolled at public universities in Egypt. A purposive sampling technique was employed to target students across nine hospitality and tourism colleges that actively used Microsoft Copilot for educational purposes. The inclusion criteria for participants were students currently enrolled in hospitality and tourism programs who had used Microsoft Copilot. Students who did not use the system at the time of the data collection were excluded.

### 3.2. Scale Measurement Development

The survey was conducted in three sections. The research goals and instructions for completing the survey are described in the first section. Details regarding the demographic characteristics of the participants are provided in the second section. The third section of this study examines several facets. The study employed a 5-point Likert scale, where 1 denoted “strongly disagree” and 5 denoted “strongly agree”. The Microsoft Copilot UTAUT scale was derived from Venkatesh (2022) and consists of four variables: EE, PE, SI, and FC. The behavioral intention (BI) scale, developed from Ajzen and Fishbein (1972), and intention to use, which was created by Venkatesh et al. (2012), are additional variables included in the Microsoft Copilot BI scale (see Appendix A). The survey was evaluated by faculty members and educational experts to ensure its consistency and ease of use. Based on the participants’ and scholars’ responses, certain statements were rewarded and rearranged to preserve the survey’s content validity. To examine the construct validity, the psychometric qualities of several scales using standards such as Cronbach’s alpha, composite reliability (CR), and average variance extracted (AVE) were part of the examination of the outer model (see Table 1). Standardized loadings “λ” as shown were above 0.7 for every scale item, indicating strong convergent validity.

### 3.3. Data Collection Procedures

The survey was conducted among hospitality and tourism students who used Microsoft Copilot for educational purposes. The instrument is available in both Arabic and English, and the translation was verified by two bilingual specialists. A pilot test involving 20 faculty members from the hospitality and tourism disciplines was conducted to ensure the clarity of the survey items. Minor revisions were made based on feedback from the pilot phase.

The online survey was distributed via email and social media platforms to students in the nine selected colleges following the recommendations of J. Hair et al. (2013). An introduction to the study, including information about its objectives, confidentiality, and participation consent, was provided, along with the survey URL. The participants were informed of the study’s ethical standards and provided verbal consent before participation. The data collection process lasted from October to December 2024. The final sample consisted of 760 valid responses from 900 distributed surveys, achieving an 84% response rate. This sample size was deemed appropriate given the item-to-sample ratio according to the criteria Nunnally and Bernstein (1994). Of the 760 valid responses, 443 respondents (58.3%) were identified as male, while 317 (41.7%) were identified as female. The age range of less than 20 to 25 years comprised the largest proportion of students, accounting for 53.1% of the responses. Within this age range, 54.4% of students were identified as junior and senior students.

The participants were informed of the study objectives to guarantee proper standards of ethical considerations. Prior to the quantitative phase of the questionnaire, they provided verbally informed consent and were assured that their answers would remain anonymous. The participants were identified by the researcher through their networks, which included friends, family, classmates, and professors. They all agreed that the data were being collected for research purposes, and that their participation was voluntary.

### 3.4. Data Analysis

Data were analyzed using partial least squares structural equation modeling (PLS-SEM), a variance-based technique suited for exploratory and predictive research. PLS-SEM was chosen because of its flexibility in handling both small and large sample sizes and its applicability without assumptions about normality (J. F. Hair et al., 2017; Do Valle & Assaker, 2016). Additionally, Henseler et al. (2009), PLS-SEM has gained popularity because of its use in exploratory and prediction-focused research. The analysis was conducted using SmartPLS 4.01 (Ringle et al., 2020). To control for common method variance (CMV), Harman’s single-factor test was performed, following Podsakoff et al. (2003). The bootstrapping approach utilized the reflecting mode, and *n* = 5000 resamples were used to estimate the model (Do Valle & Assaker, 2016). As described by Ringle et al. (2020), we implemented two phases in PLS-SEM processing: first, assessing the outside model or measuring model for construct validity and reliability, and second, assessing the inner or structural model (for hypothesis testing).

## 4. Results

The results of an exploratory factor analysis (EFA) of all 25 questions showed that the first component accounted for 35.28% of the variation. Therefore, CMV was not a major factor in this study. Furthermore, there were no multicollinearity problems because the variance inflation factor (VIF) values were less than five (Table 1).

Using Fornell and Larcker’s (1981) method to verify discriminant validity, we ensured that each construct’s square root of the average variance extracted (AVE) was greater than the correlations across it and every other construct (Table 2).

The Heterotrait–monotrait (HTMT) ratio measures the degree of similarity between latent components to evaluate discriminant validity. Henseler et al. (2016) claimed that an HTMT score of less than 0.90 proves established discriminant validity. As shown in Table 3, all HTMT ratios were below the 0.90 criterion, therefore showing well-established discriminant validity. This strengthens the dependability of the results, because the model’s constructions measure different concepts and are not closely connected.

**Figure 2:**
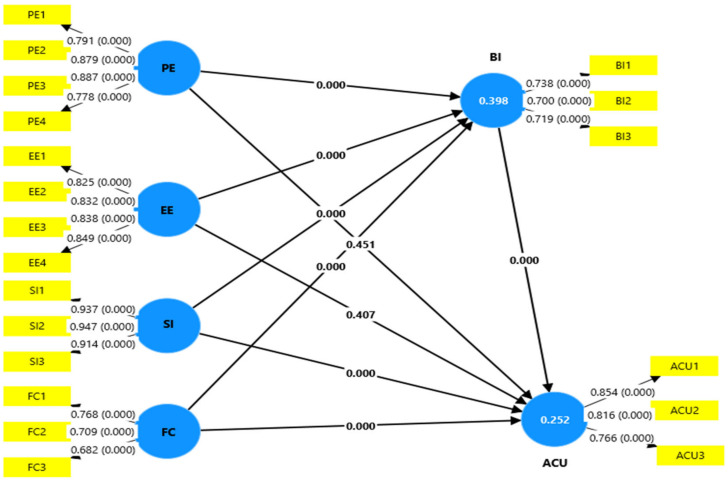
The Research Final Model.

As shown in Table 4 and Figure 2, the results supported that behavioral intention (BI) is a decisive factor for students’ perceptions regarding using Microsoft Copilot and their actual usage, supporting H1, H2, H3, and H4. The results revealed that PE significantly affected BI (β = 0.186, T = 4.588, *p* = 0.000), meaning that students intended to use a system more if they believed it to be beneficial. Moreover, EE had an important positive effect on BI (β = 0.260, T = 7.006, *p* = 0.000), and EE was an important driver of intention formation. In terms of SI, it also helps explain other influences on BI (β = 0.184, T = 4.956, *p* = 0.000), explaining that peer and social encouragement influences individuals’ adoption intentions. Additionally, FC can strongly affect BI (β = 0.246,T = 7.242, *p* = 0.000), which implies that having access to resources and support system mechanisms enhances a user’s probability of having a favorable intention toward system adoption. Surprisingly, PE (β = −0.038, T = 0.753, *p* = 0.451) and EE (β = 0.049, T = 0.829, *p* = 0.407) had non-significant effects on ACU, resulting in the rejection of H5 and H6. This indicates that students’ perceived usefulness and ease of use alone may not directly translate into the actual use of Microsoft Copilot. On the other hand, SI (β = 0.142, T = 3.529, *p* = 0.000) and FC (β = 0.174, T = 3.807, *p* = 0.000) both had a pivotal and significant impact on the actual use of Microsoft Copilot, supporting H7 and H8, indicating that social encouragement and environmental support facilitate actual involvement with the system. With the strongest effect on actual usage, the most notable was BI (β = 0.417, T = 8.166, *p* = 0.000), confirming the basis of behavioral models, which is that the primary predictor for actual behavior is intention.

Regarding the indirect effects, Table 3 reveals that BI fully mediates the relationship between PE (β = 0.078, T = 4.152, *p* = 0.000), EE (β = 0.108, T = 4.979, *p* = 0.000), and ACU, confirming H10 and H11. This implies that students who feel a system is useful and easy to use do not inherently use this system but must first gain a strong behavioral intention that drives system adoption. Moreover, BI partially mediated SI (β = 0.077, T = 4.146, *p* = 0.000) and FC (β = 0.103, T = 5.419, *p* = 0.000), and ACU supported H12 and H13 (see Table 4). Thus, social influence and available resources, individually attending to and using, also reinforce intention formation, which in turn leads to engagement. These findings demonstrate that even some (SI, FC) of these predictors have both direct and indirect effects on usage, while others (PE, EE) depend completely on BI in impacting usage. With these mediation patterns and the strong direct effect of BI on ACU, intention formation and the formation of the technology adoption bridge highlight the central role of intention formation in technology adoption. Consequently, strategies intended to boost system adoption should aim to increase users’ behavioral intentions by emphasizing its perceived usefulness, ease of use, and the influence of social and environmental factors that facilitate adoption.

## 5. Discussion and Implications

With an emphasis on hospitality and tourism students in public Egyptian universities, this study aimed to explore how higher education students accept and use Microsoft Copilot for academic purposes. The pre-test survey results, which were based on the UTAUT framework, revealed intriguing information regarding the main factors influencing the use of Microsoft Copilot for learning.

The findings confirmed the strong influence of PE and EE on BI, supporting the first and second hypothesis (H1 and H2). These results align with those of previous research (Strzelecki et al., 2024; Xia & Chen, 2024), reinforcing the idea that students intend to use AI platforms more if they believe they are beneficial and easy to use. Students are more likely to form the intention to integrate the tool into their educational activities when they feel that it is entertaining (EE) or easy to use (PE). The high influence of both PE and EE emphasizes the relevance of user-friendly engaging platforms for fostering technological acceptance. Therefore, it is imperative that policymakers and educators concentrate on developing AI tools that not only make difficult activities easier, but also improve the learning process to promote students’ continued use of them.

Additionally, the findings supported that SI is the most reliable indicator of students’ BI toward Microsoft Copilot adoption for educational purposes among Egyptian hospitality and tourism colleges, confirming the third and seventh hypotheses (H3 and H7). This result is in line with earlier research (Ma & Huo, 2023; Nik Harun et al., 2024; Pan et al., 2024; Menon et al., 2025), which showed a considerably favorable impact of SI on the utilization of Microsoft Copilot in higher education. The current research demonstrates that when classmates and colleagues provide constructive criticism regarding artificial intelligence (AI) and its educational advantages, it has a beneficial impact on other students’ behavioral desires to use this technology in the classroom (e.g., Microsoft Copilot). Moreover, SI plays a pivotal role in shaping students’ actual use of Microsoft Copilot, thus verifying H7, which is consistent with previous studies (e.g., Yin et al., 2023; Dogru et al., 2024; Sobaih et al., 2024), strengthening students’ intention to use Microsoft Copilot because their peers believe it is a useful tool and advise them to use it for academic purposes. This is evident because education is a combined effort in both its curriculum and learning environment, where peer referrals and faculty endorsements are leading factors in a student’s opinion and behavior. Within the context of AI integration in practical learning environments for hospitality and tourism higher education, there is more pressure on academic and industry leaders to implement broader, and strategized, approaches to adoption. Additionally, the employment of AI advanced academic and hands-on training can enhance education efficiency and prepare students to the industry’s changes.

According to the findings, among Egyptian hospitality and tourism students, FC is the second-highest indicator of BI to use Microsoft Copilot for academic purposes, supporting the fourth and eighth hypothesis (H4 and H8). This result is consistent with Menon et al.’s (2025) research, which also indicated that FC is a major factor in determining students’ BI to use Microsoft Copilot in the classroom. According to another, earlier study (e.g., Adetayo et al., 2024), there is a strong positive correlation between FC and BI when it comes to using Microsoft Copilot technology in the classroom. Due to the partnership between policymakers in Egyptian higher education and Microsoft Corporation, which allows students free and accessible interaction, the availability of infrastructure as well as technical support linked with students’ academic accounts will serve as a pivotal motivation for students to engage with Microsoft AI tools such as Copilot. Regarding the influence of BI on the actual use of Microsoft Copilot, the results revealed a strong and positive influence of BI on the ACU of Copilot. These results support hypothesis (H9) and align with previous research (e.g., Anditama & Hidayanto, 2024; Pan et al., 2024; Menon et al., 2025), demonstrating that students are more likely to utilize Microsoft Copilot when they have a favorable BI toward it, indicating that they are prepared to incorporate it into their daily routines or tasks.

Surprisingly, there was no evident direct impact of PE or EE on the actual use of Microsoft Copilot in the classroom, thus rejecting the fifth and sixth hypothesis (H5 and H6). These surprising results did not align with those of several studies (e.g., Anditama & Hidayanto, 2024; Kim et al., 2024; Strzelecki et al., 2024; Xia & Chen, 2024), which found that PE and EE are considered predictors of students’ BI and actual use of Microsoft Copilot for educational purposes. While PE and PE might shape students’ intention to use Copilot, the final decision to use it is often influenced by habit, necessity, or institutional factors (e.g., university requirements, course integration). Furthermore, the unexpected lack of a direct effect could be due to hospitality and tourism students not relying heavily on Microsoft Copilot for their coursework or practical tasks, even if they expect it to be useful. Additionally, despite the positive aspects of Microsoft Copilot, such as ease of use and effortlessness, students may not be motivated to use it when their classmates or educators are not prompted to promote it.

In terms of indirect effects, BI was found to fully mediate the relationship between EE, PE, SI, and FC and the actual use of Microsoft Copilot in educational institutions for learning purposes, supporting the tenth and eleventh hypothesis (H10 and H11). This finding is consistent with the UTAUT framework (Venkatesh, 2022) and Strzelecki’s (2023) findings. In particular, the relationship between PE and EE and the employment of Microsoft Copilot in the classroom was found to be fully mediated by BI. However, the relationship between SI and FC and the utilization of Microsoft Copilot for academic purposes was partially mediated, confirming the twelfth and thirteenth hypothesis (H12 and H13). This result is in line with a recent study by A. Hasanein et al. (2024), who reinforced that BI slightly shapes the relationship between SI, FC, and the actual use of AI platforms (that is, Gemini). As mentioned before, students developed a negative BI to use Microsoft Copilot due to a lack of resources and support, which had a negligible impact on their actual use of the program in the classroom. According to these findings, if hospitality and tourism colleges motivate their students to use Microsoft Copilot in their coursework, they must provide them with necessary tools and assistance.

## 6. Conclusions

This study was conducted to examine the acceptance and utilization of Microsoft Copilot for academic purposes among hospitality and tourism students in higher education, given the growing use of this advanced technology. This study enhances our understanding of the factors influencing college students’ adoption and use of Microsoft Copilot. The findings partially supported the UTAUT model and previous research, revealing significant direct effects of EE, PE, SI, and FC on the behavioral intention to use Microsoft Copilot, whereas only SI, FC, and BI had significant direct effects on its actual use. However, the results diverged from earlier studies on the minor impact of PE and EE on Microsoft Copilot usage. Consequently, BI partially mediated the relationship between SI and FC and the actual use of Microsoft Copilot in educational settings. In contrast, the relationship between BI, PE, and EE and the actual use of Microsoft Copilot in the classroom was fully mediated.

### 6.1. Study Limitations

This research was conducted on a purposive sample of hospitality and tourism students at an Egyptian public university. Interesting discoveries that both supported and opposed the UTAUT paradigm were revealed by these results. The moderating influence of participant demographics, including gender, age, specialization, study year, and prior experience with the use of technology in the classroom, was not investigated in this study. This could be another area for exploration in future studies.

### 6.2. Future Research

Although the current research offers initial knowledge of how students view Microsoft Copilot in hospitality and tourism education, numerous areas deserve further research. Future research should focus on the long-term influence of changes in AI adoption behavior. Moreover, a qualitative approach, such as interviews or focus groups with faculty members and educational leaders, could also help reveal more about students’ perceptions of adoption challenges and motives. Comparative research from multidisciplinary fields or different educational regions could uncover related variations in utilizing AI-powered technology in educational contexts.

## Figures and Tables

**Table 1:** Measurements and Variable Parametric Attributes.

Scale Variables	λ	VIF
**Performance Expectancy: (α = 0.891, CR = 0.892, AVE = 0.753)**		
PE1	0.791	1.822
PE2	0.879	2.710
PE3	0.887	2.707
PE4	0.778	1.568
**Effort Expectancy: (α = 0.795, CR = 0.825, AVE = 0.862)**		
EE1	0.825	2.023
EE2	0.832	1.756
EE3	0.838	2.090
EE4	0.849	2.351
**Social Influence: (α = 0.883, CR = 0.893, AVE = 0.862)**		
SI1	0.937	4.337
SI2	0.947	4.234
SI3	0.914	2.804
**Facilitating Conditions: (α = 0.942, CR = 0.806, AVE = 0.769)**		
FC1	0.768	1.045
FC2	0.709	1.524
FC3	0.682	1.504
**Behavioral Intention: (α = 0.793, CR = 0.762, AVE = 0.867)**		
BI1	0.738	1.207
BI2	0.700	1.118
BI3	0.719	1.132
**Actual Usage: (α = 0.856, CR = 0.959, AVE = 0.856)**		
ACU1	0.854	1.760
ACU2	0.816	1.683
ACU3	0.766	1.290

**Table 2:** Fornell and Larcker Discriminant Validity.

	BI	EE	FC	PE	SI	ACU
**BI**	**0.719**					
**EE**	0.505	**0.836**				
**FC**	0.477	0.490	**0.721**			
**PE**	0.437	0.430	0.325	**0.835**		
**SI**	0.366	0.243	0.238	0.323	**0.933**	
**ACU**	0.456	0.294	0.351	0.176	0.052	**0.813**

Bold ratios show the square root of AVE.

**Table 3:** Heterotrait-Monotrait (HTMT) Discriminant Validity.

	BI	EE	FC	PE	SI	ACU
**BI**						
**EE**	0.735					
**FC**	0.786	0.599				
**PE**	0.642	0.501	0.434			
**SI**	0.521	0.270	0.317	0.361		
**ACU**	0.725	0.362	0.536	0.248	0.068	

**Table 4:** Direct and indirect path coefficients.

Paths	Path Coefficient	T Value	*p* Values
**Direct Effect**			
**[H1]** PE ➜ BI.	0.186	4.588	0.000
**[H2]** EE ➜ BI.	0.260	7.006	0.000
**[H3]** SI ➜ BI.	0.184	4.956	0.000
**[H4]** FC ➜ BI.	0.246	7.242	0.000
**[H5]** PE ➜ ACU.	−0.038	0.753	0.451
**[H6]** EE ➜ ACU.	0.049	0.829	0.407
**[H7]** SI ➜ ACU.	0.142	3.529	0.000
**[H8]** FC ➜ ACU.	0.174	3.807	0.000
**[H9]** BI ➜ ACU.	0.417	8.166	0.000
**Indirect Effect**			
**[H10]** PE ➜ BI ➜ ACU.	0.078	4.152	0.000
**[H11]** EE ➜ BI ➜ ACU.	0.108	4.979	0.000
**[H12]** SI ➜ BI ➜ ACU.	0.077	4.146	0.000
**[H13]** FC ➜ BI ➜ ACU.	0.103	5.419	0.000

## Data Availability

Data are available upon request from researchers who meet the eligibility criteria. Kindly contact the first author privately through e-mail.

## List of Acronyms

**Acronym**	**Full form**
AI	Artificial Intelligence
BI	Behavioral Intention
UTAUT	Unified Theory of Acceptance and Use of Technology
EE	Effort Expectancy
PE	Performance Expectancy
SI	Social Influence
FC	Facilitating Conditions

## Appendix A

### Research Measurement Scale

**Study Constructs**	**Measurement Scale Items**
Performance Expectancy (PE)	PE1: “I find Microsoft Copilot to be a useful tool for my academic activities.”
	PE2: “By using Microsoft Copilot, you have a better chance of accomplishing important academic objectives.”
	PE3: “By accelerating task and project completion, Microsoft Copilot increases academic productivity.”
	PE4: “I can improve my academic performance by using Microsoft Copilot.”
Effort Expectancy (EE)	EE1: “I find Microsoft Copilot to be easy to learn.”
	EE2: “The exchanges with Microsoft Copilot are clear and understandable.”
	EE3: “Microsoft Copilot is intuitive and easy to use.”
	EE4: “I find it easy to become proficient with Microsoft Copilot.”
Social Influence (SI)	SI1: “My close peers, believe that I should use Microsoft Copilot.”
	SI2: “My classmates shape my behavior recommend the utilization of Microsoft Copilot.”
	SI3: “I’m advised to utilize Microsoft Copilot by people whose opinions I respect.”
Facilitating Conditions (FC)	FC1: “I possess the resources I need to utilize Microsoft Copilot.”
	FC2: “I have learnt how to use Microsoft Copilot, so I am proficient in it.”
	FC3: “My technology is compatible with Microsoft Copilot.”
	FC4: “External resources can provide support and assistance when encountering issues with Microsoft Copilot.”
Behavioral Intention (BI)	BI1: “I decided to keep using Microsoft Copilot in the future.”
	BI2: “I’m committed to using Microsoft Copilot as an academic tool.”
	BI3: “My goal is to keep using Microsoft Copilot regularly.”
Actual Use (ACU)	ACU1: “I want to apply the information and abilities I gained from the Microsoft Copilot to my academic achievements.”
	ACU2: “I find that the information and abilities I gained from the Microsoft Copilot will be helpful in the classroom.”
	ACU3: “Using Microsoft Copilot has helped to improve my academic performance.”
